# Effects of Konjac Glucomannan and Curdlan on the 3D Printability and Physicochemical Properties of Germinated Brown Rice Gel

**DOI:** 10.3390/foods14101764

**Published:** 2025-05-16

**Authors:** Chun Bai, Ran Liu, Liuyang Shen, Yu Zhuang, Jiaying Hu

**Affiliations:** 1 College of Arts and Sciences, Northeast Agricultural University, Harbin 150030, China; 2 College of Engineering, Northeast Agricultural University, Harbin 150030, China

**Keywords:** 3D printing, germinated brown rice, hydrocolloids, food manufacturing

## Abstract

Germinated brown rice (GBR), rich in high starch content and bioactive compounds, has excellent gel-forming properties, rendering it highly promising for applications in food 3D printing, a cutting-edge personalized manufacturing technology. This study systematically investigates the effects of different concentrations of konjac glucomannan (KGM) and curdlan (CD) blends on the 3D printing performance and physicochemical properties of GBR gel. The results indicated that the appropriate addition of KGM/CD blends significantly enhances the printing accuracy and shape retention of GBR gel. Specifically, under the KGM to CD ratio of 3:1 (KC3) formulation obtained by combining 2.25% KGM and 0.75% CD, the printing accuracy was highest with a minimized error of 4.97 ± 0.45%, and optimal structural stability was maintained within 5 h post-printing. Rheological measurements revealed that the flow behavior index (*n*) of the KC3 system was 0.049 ± 0.014, indicating superior flowability and significantly improved overall rheological stability. Additionally, the blend system not only increased the hardness and gel elasticity of the GBR gel but also significantly enhanced its cohesiveness and adhesiveness, reaching the highest values of 0.323 ± 0.02 and −217.488 ± 22.499, respectively, in the KC3 formulation. Further thermal analysis, low-field nuclear magnetic resonance analysis, along with Fourier-transform infrared spectroscopy and scanning electron microscopy observations, collectively demonstrated that the KGM/CD blend effectively reinforced the stability of the GBR gel network structure. These findings provide theoretical support for optimizing GBR applications in food 3D printing.

## 1. Introduction

Three-dimensional (3D) printing technology, with its advantages of high efficiency, low cost, and high customizability, has been widely applied in various fields, including industrial manufacturing, mechanical engineering, and biomedical sciences [1]. As an innovative food processing technology based on additive manufacturing, food 3D printing primarily employs extrusion-based, inkjet-based, and binder-based printing techniques to achieve precise structural construction through the layer-by-layer deposition of materials [2,3]. With the advancement of the economy and society, consumer demand for food has evolved beyond singular flavors, requiring innovations in sensory experience. 3D food printing eliminates the dependence on uniquely shaped molds and maximizes design possibilities [4]. It enables the use of diverse materials to create complex geometric structures that are challenging to achieve with traditional processing methods, thereby elevating food to new levels in terms of aesthetics, texture, and functionality [5].

Germinated brown rice (GBR) has garnered significant attention in functional food research due to its rich nutritional profile and potential health benefits. It is abundant in dietary fiber, phenolic compounds, and various bioactive components, demonstrating significant advantages in improving metabolic syndrome, enhancing neuroprotective effects, and modulating gut microbiota. These attributes confer GBR with high nutritional value and promising edible potential [4,6,7,8]. Starch, as one of the most commonly used structural matrices in food 3D printing, plays a crucial role in optimizing rheological properties, enhancing printing stability, and improving product texture due to its excellent gelling properties and shear-thinning behavior [9]. Previous studies have reported the utilization of corn starch [10], potato starch [11], and wheat starch [12] in 3D food printing. While starch-based 3D printing has been widely explored, studies focusing on GBR remain extremely limited. However, despite its rich starch content, GBR alone is limited in 3D printing applications, as starch-based inks often exhibit high viscosity, stickiness, and weak self-supporting ability, resulting in poor shape fidelity [13]. To enhance printability, food hydrocolloids such as konjac glucomannan (KGM) and curdlan (CD) are commonly added to starch-based material to formulate the composite gels.

Hydrocolloids are widely used as rheological modifiers and structural support materials in food 3D printing. Numerous studies have demonstrated that hydrocolloids are among the most critical additives in this field [14,15,16]. KGM is a linear random copolymer composed of β-1,4-linked D-mannose and D-glucose in a molar ratio of 1.6:1. It can form gels at high concentrations; however, its gelation has certain limitations, such as requiring a high KGM content and producing an undesirable texture [17]. CD is a linear polysaccharide composed of repeating (1 → 3)-β-D-glucose units and exists in three conformations: single-helix, triple-helix, and random coil. Its gelation behavior is significantly influenced by the heating conditions, forming a reversible low gel at 55–65 °C, which transitions into an irreversible high gel upon heating at 80–130 °C [18]. Various hydrophilic colloids have been widely used in the field of food 3D printing, and blending hydrocolloids has been shown to significantly enhance gel performance. Kim et al. [19] developed 3D-printed rice flour foods suitable for individuals with dysphagia using a κ-carrageenan–curdlan blend. Yu et al. [16] improved the printability of fish paste through a konjac gum–xanthan gum mixture. Feng et al. [20] demonstrated that combining agar with konjac gum enhanced the elasticity and shape fidelity of printed foods. Wu et al. [21] found that the synergistic interaction between KGM and CD can further optimize rheological properties. This synergistic effect not only improves the rheological properties of printing materials but also enhances their printing accuracy and structural retention, demonstrating the broad application potential of composite hydrocolloids in food 3D printing.

This study aimed to develop a 3D-printable gel system based on GBR by incorporating food-grade hydrocolloids to improve its printing performance. In the preliminary stage, five common hydrocolloids (xanthan gum, sodium carboxymethyl cellulose, pectin, konjac glucomannan, and curdlan) were tested, and simple combinations of selected hydrocolloids were also evaluated to assess their effects on the quality of the GBR gel. The results showed that KGM and CD, particularly at a 3% concentration, significantly improved the gel texture, stability, and rheological behavior. Therefore, KGM and CD were selected as the composite hydrocolloids for further optimization of the GBR gel system. This study focused on analyzing the physicochemical properties, microstructure, and 3D printability of the GBR-KGM/CD composite gel. By expanding the potential applications of GBR and leveraging 3D printing technology, this research provides theoretical support and practical guidance for the development of GBR foods.

## 2. Materials and Methods

### 2.1. Preparation of GBR Powder

The preparation of GBR was conducted with reference to Zhu et al. [8] with slight modifications. After the germination process, the GBR was immediately freeze-dried using a lyophilizer (Boyikang Experimental Instruments Co., Ltd., Beijing, China) until the moisture content was reduced to 8.0 ± 0.5% (w.b). The freeze-dried GBR was then ground using a pulverizer (Leimai Machinery Equipment Co., Ltd., Guangzhou, China) and passed through a 100-mesh sieve to obtain uniformly sized GBR powder.

### 2.2. Preparation of GBR Gel

**Figure 1:**
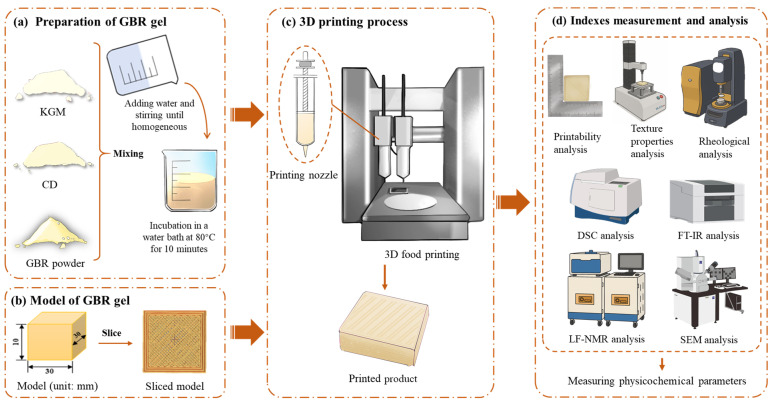
Flowchart of GBR gel preparation and its 3D printing process.

The GBR gel was prepared following the method described by Huang [22] with appropriate modifications. The flowchart of the gel preparation and 3D printing process is shown in Figure 1, and the experimental formulations of the GBR gel for 3D printing is shown in Table 1. The freeze-dried GBR powder and hydrocolloids were first mixed with boiled distilled water (98.0 ± 0.5 °C) at a solid-to-liquid ratio of 1:3 (*w*/*w*). The mixture was homogenized using a high-speed mixer for 5 min until fully blended, and then incubated in a water bath (Zhiborui Instrument Manufacturing Co., Ltd., Changzhou, China) at 80 °C for 10 min to promote gelation, with a plastic film covering the surface to prevent moisture loss. Finally, the samples were cooled to room temperature, sealed with plastic wrap, and stored at 4 °C for 24 h before further analysis.

### 2.3. Three-Dimensional Printing Process of GBR Gel

A cuboid 3D printing model (length 30 mm × width 30 mm × height 10 mm) was pre-designed using SolidWorks 2024 software, and the corresponding printing path was generated via Slic3r software (version 1.3.0). The food 3D printer (Shiyin Technology Co., Ltd., Hangzhou, China) was configured with the following parameters: nozzle diameter of 1.2 mm, infill density of 60%, nozzle movement speed of 25 mm/s, and layer height of 0.9 mm. All printing processes were conducted under controlled ambient conditions at 25 ± 1 °C.

### 2.4. Evaluation of 3D Printability

Fidelity: Following the completion of the printing process, images of the printed samples were captured in a controlled black-box environment. The central length, width, and height of the samples were measured using a right-angle ruler. The printing deviations in the central length, width, and height (*D_L_*, *D_W_*, *D_H_*) were determined using Equation (1), while the overall geometric deviation (*D_C_*) was calculated using Equation (2). A lower deviation value indicates superior printing accuracy and precision [23]:

(1) $$\mathrm{Printing}\;\mathrm{Deviation}\left(\%\right)=\left(\frac{\left|D_{M}\right|}{D_{0}}-1\right)\times 100\%$$

(2) $$D_{C}\left(\%\right)=(D_{L}+D_{W}+D_{H})/3$$

where *D_M_* represents the actual dimensions of the printed sample, while *D*_0_ denotes the standard dimensions of the model. The parameters *D_L_*, *D_W,_* and *D_H_* indicate the respective deviations in the length, width, and height, while *D_C_* represents the overall geometric deviation in the printed sample.

Structural Stability: The dimensional stability of the printed samples was assessed over a period of 6 h. The central length, width, and height of each sample were measured at hourly intervals. The structural stability deviation was quantified by stability index (*D_S_*) using Equation (3) [23]:

(3) $$D_{S}\left(\%\right)=\left(D_{LH}+D_{WH}+D_{HH}\right)/3$$

where *D_S_* represents the stability index at each time point, and *D_LH_*, *D_WH,_* and *D_HH_* correspond to the deviations in the length, width, and height at each hourly measurement, as determined by Equation (1).

### 2.5. Texture Profile Analysis (TPA)

The textural properties of the printed samples were evaluated using a texture analyzer (TA Instruments, Inc., New Castle, DE, USA) operating in Texture Profile Analysis (TPA) mode. A cylindrical probe with a diameter of 50 mm (P/50) was employed for the measurements. The instrument was configured with a 5 kg load cell and a trigger force of 5 g. The samples were compressed to 45% of their original height, with the test parameters set as follows: pre-test speed of 1 mm/s, test speed of 5 mm/s, and post-test speed of 5 mm/s.

### 2.6. Rheological Characterization

Rheological properties were analyzed using a rheometer (TA Instruments, Inc., New Castle, DE, USA) equipped with a parallel plate geometry (diameter: 40 mm; gap: 1000 μm). The experimental protocol was adapted from established methodologies [22] with necessary modifications. For dynamic rheological analysis, frequency sweep tests were performed over a range of 0.1–10 Hz at a constant strain of 2% and a controlled temperature of 25 °C. The storage modulus (G′) and loss modulus (G″) were recorded as functions of the frequency to evaluate the viscoelastic properties of the gel system. The loss tangent (tan δ), defined as the ratio of G″ to G′, was calculated using Equation (4) [24]:

(4) $$\tan\delta =\frac{G^{\prime \prime }}{G^{\prime }}$$

For shear rheology, the shear rate was varied from 0.1 to 10 rad/s to obtain the flow behavior curves, denoting the shear stress and viscosity as functions of the shear rate. The obtained shear curves were fitted through the power-law model using Equation (5) [25]

(5) $$\eta =K\gamma^{n-1}$$

where *η* denotes the viscosity (Pa·s), *γ* represents the shear rate (s^−1^), *K* is the consistency coefficient (Pa·s), and *n* is the flow behavior index of the power-law model.

### 2.7. Low-Field Nuclear Magnetic Resonance (LF-NMR)

The water distribution and relaxation time (T_2_) of the composite gel were measured using an LF-NMR analyzer (Niumag Co., Ltd., Suzhou, China), and the LF-NMR measurement of the sample was conducted according to the method described by Yu et al. [8] with slight modifications.

### 2.8. Differential Scanning Calorimetry (DSC)

The gelatinization properties of the GBR gels were analyzed using DSC (Model Q20, TA Instruments Inc., New Castle, DE, USA). An amount of 5 mg of the gel sample was accurately weighed and transferred into an aluminum pan, followed by equilibration at room temperature for more than 4 h. The sample was heated at a rate of 10 °C/min within a scanning temperature range of 20–100 °C, using a sealed empty aluminum pan as a reference. The gelatinization temperature and enthalpy changes were recorded, and the onset gelatinization temperature (*T*_0_), peak gelatinization temperature (*T_p_*), conclusion gelatinization temperature (*T_c_*), and enthalpy change (Δ*H*) were calculated.

### 2.9. Fourier-Transform Infrared Spectroscopy (FT-IR)

The gel samples were freeze-dried using a lyophilizer, ground into powder, and stored in a desiccator (IRAffinity-1S, Shimadzu, Kyoto, Japan). The potassium bromide (KBr) pellet method was employed, in which 1 mg of the sample was mixed with 100 mg of KBr powder, ground, and pressed into a thin disc. The infrared spectra were recorded in the wavenumber range of 400–4000 cm^−1^ with a resolution of 4 cm^−1^.

### 2.10. Scanning Electron Microscopy (SEM)

The gel samples were freeze-dried by a lyophilizer and then completely mounted onto the observation platform. A vacuum sputter coater was used for gold sputtering under vacuum conditions. SEM (S-3400 N, Hitachi, Tokyo, Japan) was performed at an accelerating voltage of 5 kV, with images captured at a magnification of 1000×.

### 2.11. Data Analysis

All statistical analyses were performed using SPSS 26.0 software (SPSS Inc., Chicago, IL, USA). One-way analysis of variance (ANOVA) and Duncan’s multiple range test were conducted, with statistical significance set at *p* < 0.05. Experimental results were visualized using Origin 2024 software (OriginLab Corp., North Hampton, NH, USA).

## 3. Results and Discussion

### 3.1. Evaluation of 3D Printability Performance of GBR Gel

The effects of different formulations on the 3D printing performance of the GBR gel are summarized in Table 2. The control group exhibited extremely poor printability, with uneven extrusion lines. This issue primarily arose due to shear-induced flocculation occurring when the fiber-rich gel passed through the nozzle under the applied pressure of the printer, ultimately leading to nozzle clogging [26]. In contrast, the addition of KGM and CD significantly improved the printing performance of the gel. However, while the KGM group exhibited good structural support, the printed lines were rough, prone to trailing, and susceptible to curling at the edges. CD, known for its excellent water absorption capacity, facilitated the formation of smooth extrusion lines [18]; however, the samples demonstrated poor mechanical integrity, uneven structures, and were highly prone to collapse and deformation. The KGM-CD-GBR composite gel system not only improved the structural stability of the printed samples compared to the control and CD groups but also enhanced the overall printing fidelity relative to the KGM group.

**Figure 2:**
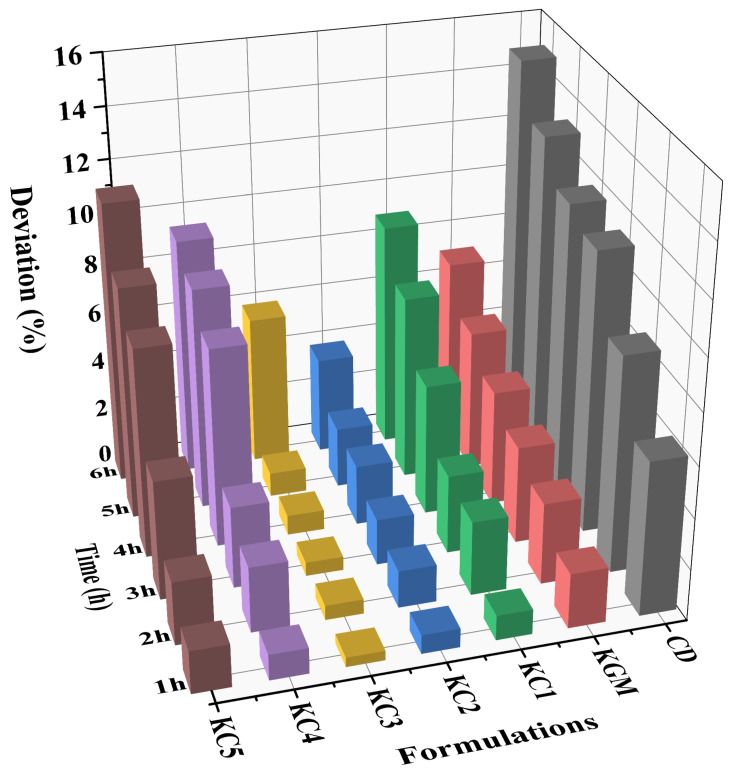
Stability variation in the printed samples among different gel formulations. Note: The meanings of the symbols for gel formulations are detailed in Table 1.

The structural stability results of the printed samples are presented in Figure 2. The CD group exhibited the lowest stability, likely due to the severe dehydration and shrinkage effects caused by high CD concentrations [27]. Within the composite hydrocolloid systems, the KC4, KC5, and KC1 groups demonstrated improved stability compared to the CD group. Notably, KC2 and KC3 demonstrated significantly enhanced stability during the first 5 h, with KC3 exhibiting the greatest overall stability. This effect can be attributed to the strong hydrogen bonding interactions between KGM and low concentrations of CD [21], coupled with the ability of starch molecules to mitigate the dehydration-induced shrinkage and syneresis of the CD gel network [28], thereby maintaining the structural integrity of the printed samples. However, after 6 h of observation, the KC3 group exhibited noticeable swelling, leading to a sharp increase in structural deviation. To address this issue, previous studies have proposed potential strategies to enhance gel stability, such as (1) employing freeze–thaw processing to form a dense dual-network structure and increase molecular flexibility, thereby improving gel stability [29]; and (2) adjusting the pH of the gel system to strengthen the intermolecular interactions, which can further enhance shape retention during long-term storage [30]. These approaches could potentially be applied in future work to extend the structural stability of GBR-based gels in 3D printing. Overall, the hold time of 3D-printed GBR gel should not exceed 5 h to ensure the structural integrity and surface accuracy of printed products.

### 3.2. Analysis of Rheological Properties of GBR Gel

**Figure 3:**
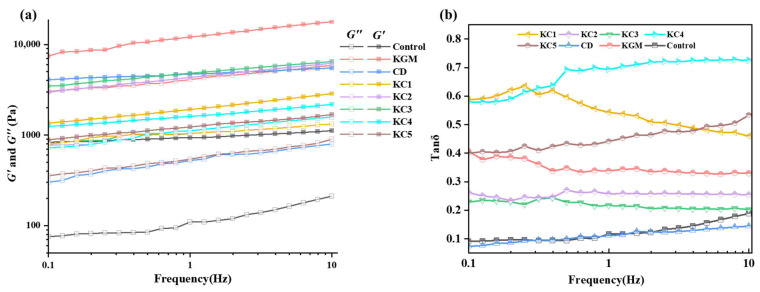
Rheological properties of GRB gel with different formulations. (**a**) Storage modulus (G′) and loss modulus (G″); (**b**) rheological loss factor (tan δ). Note: The meanings of the symbols for gel formulations are detailed in Table 1.

**Figure 4:**
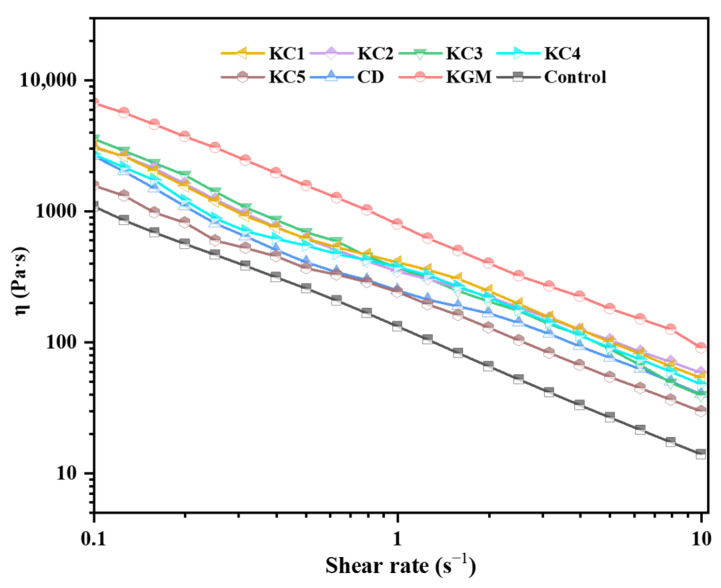
Apparent viscosity versus shear rate profile for GBR gel with different formulations. Note: The meanings of the symbols for gel formulations are detailed in Table 1.

Rheological properties play a crucial role in determining the molding quality and structural stability of 3D-printed food products. Figure 3 and Figure 4 show the rheological properties of the GRB gel with different formulations.

Figure 3a presents the elastic modulus (*G*′) and viscous modulus (*G*″) of different samples. As observed, both *G*′ and *G*″ increased with the frequency, with G′ consistently exceeding G″, indicating that, regardless of the additive incorporation, the GBR gels exhibited weak gel characteristics [31] and possessed a certain degree of self-supporting ability. Low *G*′ values are generally associated with poor shape retention capacity [3], which can lead to irregular edges or structural collapse in printed cubic structures. All additive-containing groups exhibited higher *G*′ values than the control group, suggesting synergistic interactions between the additives and the GBR gel. High concentrations of KGM significantly enhanced the mechanical strength of the samples, attributed to phase separation caused by the incompatibility between KGM and starch in the continuous phase. This phase separation effectively increased the local starch concentration by immobilizing water molecules, thereby promoting the formation of a stronger gel network [32]. As the CD concentration increased from 0.75% to 2.25%, *G*′ exhibited a decreasing trend due to interactions between CD and starch molecules, which reduced the available binding sites for KGM within the gel system. Excessively high *G*′ values can result in excessive elasticity, which may hinder smooth extrusion. Therefore, to achieve optimal printability, *G*′ must be maintained within an appropriate range. Figure 3b presents the rheological loss factor (*Tan δ*). When *Tan δ* < 1, the system exhibits elasticity-dominated characteristics. At the initial stage of CD addition, the *Tan δ* of the composite system decreased, indicating that an appropriate amount of CD enhanced the structural stability of the gel. However, as the CD concentration further increased, *Tan δ* also increased, suggesting that excessive CD addition led to structural instability, making the printed samples more prone to collapse. Therefore, in the optimization of 3D food printing formulations, the ratio of hydrocolloid combinations must be carefully controlled to ensure favorable rheological properties and printability.

Figure 4 illustrates the apparent viscosity of the gel systems at low shear rates. During the 3D printing process, the viscosity of the gel must be sufficiently low under high shear stress to ensure smooth extrusion, while it should be high enough post-deposition to support the layer-by-layer construction of the three-dimensional structure [33]. The experimental results indicated that all the GBR gels exhibit shear-thinning behavior, characteristic of pseudoplastic non-Newtonian fluids. The KGM group had the highest apparent viscosity, which, despite its superior support capacity, was beneficial for structural stability (as evidenced in the 3D printability analysis in Section 3.1), resulting in extrusion difficulties and rough printing surfaces, ultimately affecting the aesthetic quality of the printed product. The addition of CD reduced the apparent viscosity of the composite system, likely due to the formation of a network structure via intermolecular hydrogen bonding and physical entanglement of CD’s elongated fibers. Under shear stress, the rigid triple-helix conformation of CD was progressively disrupted [34], enhancing material fluidity.

Table 3 summarizes the fitting results of the apparent viscosity curves based on the power-law model shown in Equation (5). The *n* is commonly used to evaluate the flow characteristics of materials. Newtonian fluids typically have an *n* value close to or equal to 1, while lower *n* values indicate enhanced pseudoplastic behavior, signifying a more pronounced shear-thinning effect [35]. All samples exhibited *n* values below 1, with KC3 demonstrating the lowest value of 0.049 ± 0.014, indicating optimal flow properties conducive to uniform extrusion, ultimately enhancing the smoothness and surface texture of the printed lines. Some studies suggest that the consistency coefficient (*K*) is closely related to the printing process, as excessively high *K* values can increase the extrusion difficulty and even cause gel line breakage [35]. The KGM group exhibited the highest *K* value, leading to rough and uneven printed lines. However, the incorporation of CD significantly reduced the *K* value of the composite system, particularly in the KC5 group, where it dropped to 216.337 ± 36.019. This reduction is attributed to the strong interactions between KGM and CD, which increased the molecular flexibility and mobility. A similar phenomenon was reported by Kim et al. [19] in their study on the effects of κ-carrageenan and gellan gum on the rheological properties of rice cake formulations. Additionally, these findings align with the conclusions drawn from Figure 4, confirming the viscosity-reducing properties of CD, which effectively optimized the gel extrusion performance. However, excessive CD addition increased the *n* value of the gel system, thereby weakening its shear-thinning characteristics. Therefore, reasonable control of the CD concentration is necessary. The correlation coefficients (*R*^2^) of all the fitted equations exceeded 0.956, indicating a high degree of agreement between the experimental data and the fitted models [31].

### 3.3. Texture Properties Analysis of the 3D-Printed Product

**Figure 5:**
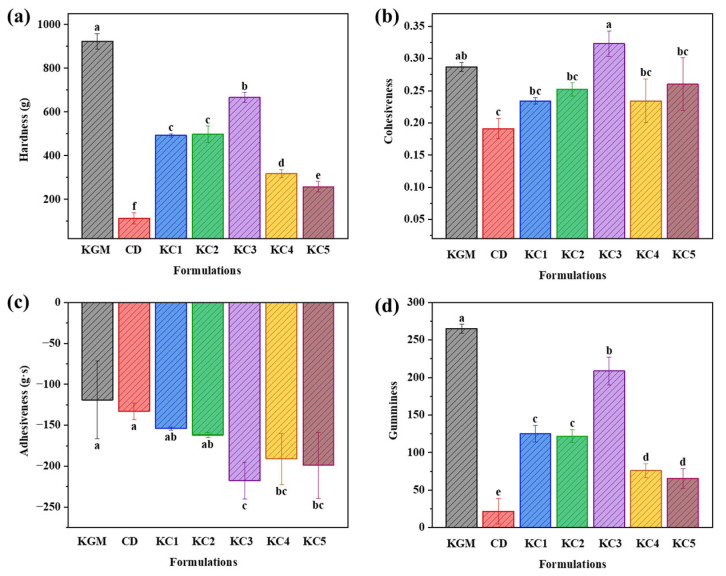
Texture properties of the 3D-printed samples with different gel formulations. (**a**) Hardness, (**b**) cohesiveness, (**c**) adhesiveness, and (**d**) gumminess. The meanings of the symbols for gel formulations are detailed in Table 1. Different letters indicate statistically significant differences (*p* < 0.05).

Texture is a key indicator of gel performance, encompassing attributes such as hardness, cohesiveness, adhesiveness, and gumminess [36]. The type and proportion of hydrocolloids significantly impact the textural properties of gels. In this study, different gel formulations were used for 3D printing, and the texture profile of the printed cubic samples was analyzed, as shown in Figure 5.

The higher the hardness of the gel, the better it can maintain the initial shape of the printed sample [37]. As shown in Figure 5a, the GBR gel containing only CD exhibited extremely low hardness, possibly because an excessive amount of CD disrupted the formation of an orderly gel matrix, thereby reducing its mechanical strength [38,39]. With increasing KGM content, the sample hardness increased significantly. This may be attributed to the strong hydrogen bonding interactions between KGM and CD molecules, which facilitated the formation of a denser gel network and enhanced interactions with the starch matrix, ultimately forming a high-strength three-dimensional structure that improved the mechanical properties of the gel system. KGM played a decisive role in enhancing the hardness of the KGM-CD-GBR gel system. It is worth noting that although previous studies reported higher gel hardness values using different substrate systems, the corresponding 3D printing performance was not necessarily superior [9]. This discrepancy may stem from differences in material composition, rheological behavior, or gelation mechanisms. In contrast, our gel system achieved a favorable balance between mechanical strength and printability, as supported by the successful extrusion and structural integrity of the printed samples. Despite the highest hardness observed in the KGM group, excessive mechanical strength may lead to nozzle clogging, resulting in rough extrusion lines or even extrusion failure, which aligns with the observation in Table 2.

Cohesiveness reflects the internal bonding strength and density of the microstructure, which helps explain the strength of internal interactions within the sample. Higher cohesiveness indicates stronger interactions within the gel, which can reduce macroscopic structural collapse caused by water loss [40]. As shown in Figure 5b, the CD group exhibited low cohesiveness, suggesting that the single-helix structures in high-concentration curdlan did not interact significantly with starch molecules, failing to form a high-strength gel. Consequently, the printed samples had a loose structure and were prone to damage. However, after adding KGM, the cohesiveness of the gel system significantly improved, with KC3 showing the highest cohesiveness (*p*< 0.05) at a peak value of 0.323 ± 0.02.

Adhesiveness measures the ability of the material to adhere to external objects [41]. Higher adhesiveness helps maintain the moisture content of the gel system. Figure 5c shows the changes in adhesiveness across different formulations. The hydrocolloid composite system reduced volume shrinkage caused by water evaporation, thereby preserving the integrity of the printed samples. The combination of KGM and CD significantly enhanced the adhesiveness of the gel system. Notably, in the KC4 and KC5 groups, the adhesiveness decreased with the increasing KGM content, but when the KGM/CD ratio reached 3:1 (KC3), the adhesiveness reached its maximum value. This may be due to the strong hydrogen bonding interactions between KGM and curdlan molecules [21].

As shown in Figure 5d, KGM exhibited the highest gumminess, while CD had the lowest. When KGM and CD were combined, the gumminess of the gel system increased with the rising KGM content, which was consistent with the hardness results. This may be because at low concentrations, the interaction sites between KGM and CD were limited, resulting in low gumminess. As the KGM concentration increased, more binding sites formed between the two components, creating a high-strength gel network and enhancing the gumminess of the gel system.

### 3.4. LF-NMR Analysis of Water Migration and Distribution

**Figure 6:**
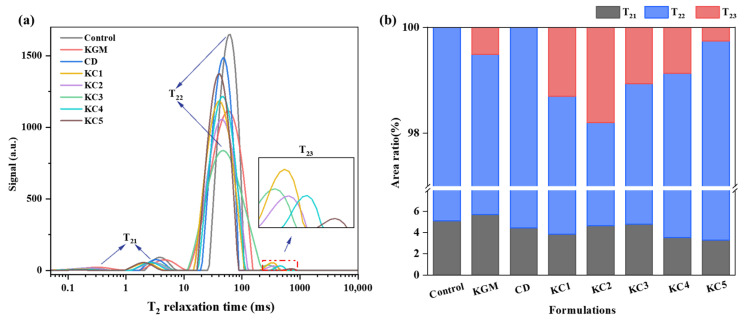
Moisture migration and distribution characteristics of GBR gel with different gel formulations. (**a**) Transverse (*T*_2_) relaxation time spectra indicating water migration behavior; (**b**) proportional distribution of water populations within the gel matrix. The meanings of the symbols for gel formulations are detailed in Table 1.

The T_2_ relaxation time provides insights into water migration and distribution within the gel system. The relaxation times of different gel samples are shown in Figure 6a, while Figure 6b presents the proportions of different types of water in each formulation. Three types of water were identified: T_21_, T_22_, and T_23_, corresponding to bound water, weakly bound water, and free water, respectively. Compared to the control group, the T_22_ relaxation time of samples containing hydrocolloids shifted closer to zero, indicating stronger interactions between water and starch, forming a denser gel network [42]. The relaxation time of the composite gels was shorter than that of the gels with a single additive, suggesting that KGM-CD had a stronger structuring effect on the gel system. Changes in water distribution directly affected the gel’s microstructure and 3D printing performance [43]. T_21_ represents water with minimal mobility, reflecting the degree of binding between polysaccharides and water molecules. The high hydrophilicity of KGM increased the bound water content, improving the gel system’s stability. However, as the CD content increased, the T_21_ proportion decreased, indicating a transition from bound water to weakly bound water. This reduced the intermolecular interactions, thereby decreasing the gel strength. T_22_ accounted for the largest proportion of water in all samples, exceeding 93.76%, highlighting its dominant role in the gel’s rheological properties [44]. The control group and KC5 had the highest T_22_ content, but their low mechanical strength and G′ values prevented the extrusion of continuous, uniform lines. As the KGM content increased, T_22_ showed a decreasing trend, but in the KC3 group, it rebounded. This may be due to KGM’s strong hydrophilicity, which led to tighter interactions between KGM, starch, and water molecules, increasing the weakly bound water fraction.

T_23_ (free water fraction): Lower free water content indicates a higher water retention capacity [45]. CD exhibited strong water-holding ability, so T_23_ increased as the CD content decreased. However, in the KC3 group, the free water percentage significantly declined, likely because starch, with its strong permeability, filled the network structure formed by high concentrations of KGM and CD, reducing the free water content [46]. Although the KC4 and KC5 groups had a lower free water content than KC3, their T_23_ relaxation times (714.943 ms and 541.587 ms, respectively) were significantly longer than KC3 (289.942 ms). This indicates weaker intermolecular bonding within the gel, and the extremely low T_21_ proportion further contributed to unstable printed structures, leading to line breakage, stretching deformation, and collapse—findings consistent with the 3D printing performance and rheological test results.

### 3.5. DSC Analysis

**Figure 7:**
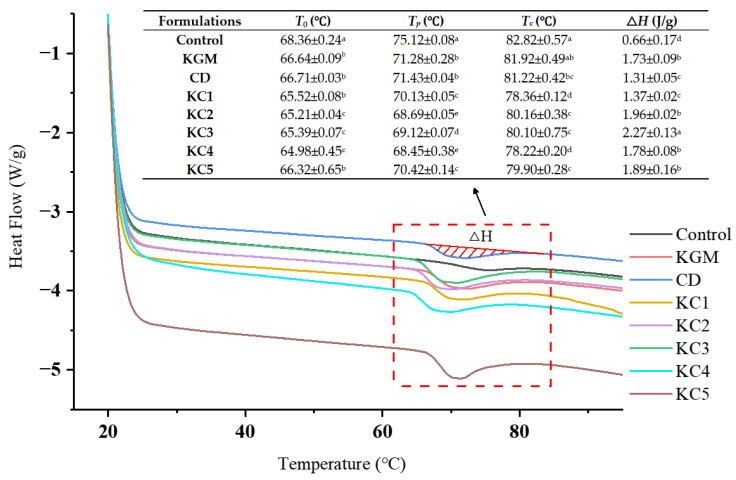
The measurement results of DSC and thermograms of different gel samples. *T*_0_: onset temperature; *T_p_*: peak temperature; *T_c_*: conclusion temperature; Δ*H*: enthalpy change. The meanings of the symbols for gel formulations are detailed in Table 1. Different letters indicate statistically significant differences (*p* < 0.05).

The DSC results are presented in Figure 7. Samples containing hydrocolloids exhibited slightly lower *T*_0_, *T_p_*, and *T_c_* compared to the control group. This suggests that, during the initial stages of gelatinization, the competition for water between hydrocolloids and starch granules was not substantially different. In contrast, Δ*H* significantly increased in all hydrocolloid-containing groups compared to the control. This may be attributed to the water-binding capacity of hydrocolloids, which restricted the availability of free water, thereby delaying starch gelatinization. As a result, the energy required for gelatinization increased, slowing down the overall gelatinization process and enhancing the thermal stability of the starch system [47].

### 3.6. FT-IR Analysis of GBR Gel

**Figure 8:**
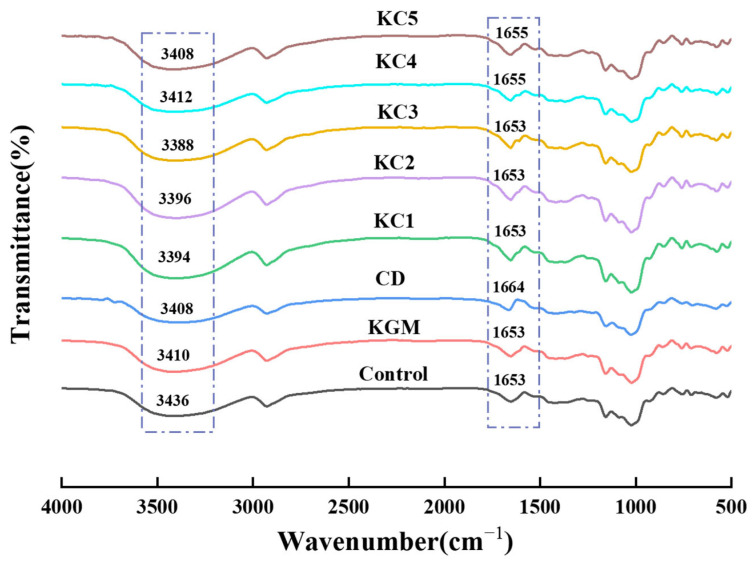
Fourier-transform infrared (FT-IR) spectral shifts of different hydrocolloids. The meanings of the symbols for gel formulations are detailed in Table 1.

Figure 8 illustrates the FT-IR spectra of the control group, KGM, CD, and their different ratio mixtures in the GBR gel within the range of 400–4000 cm^−1^. According to the spectra, the main chemical bond types among the different formulations are similar, and no new functional groups were observed. The peak around 2927 cm^−1^ corresponds to the CH_2_ asymmetric stretching vibration, while the 1655 cm^−1^ infrared absorption peak is related to the C=O stretching vibration in starch and the N-H bending vibration [8]. The 1157 cm^−1^ peak is attributed to the C–O–C groups in polysaccharides [48]. Additionally, the peaks around 940 cm^−1^ and 576 cm^−1^ represent asymmetric ring vibrations in starch, corresponding to the α-1,4-glycosidic bond skeletal vibration [12]. A broad band centered around 3500–3200 cm^−1^, associated with the O-H stretching vibration of carboxyl groups in starch, is observed in the spectra [49]. When KGM is mixed with CD, the hydroxyl vibration peak of the composite exhibits a blue shift. The binary mixtures show more pronounced shifts in KC3, KC2, and KC1, with values of 3388, 3396, and 3394 cm^−1^, respectively, compared to KGM or CD alone, and similar findings were also reported by Zhou et al. [50]. This suggests that curdlan micelles or triple-helix structures dissociate at high temperatures, exposing more molecular binding sites, which leads to stronger hydrogen bonding interactions between KGM and CD [51]. Moreover, the 1655 cm^−1^ peak represents carboxyl groups [52]. A shift toward a higher wavenumber shift in this peak suggests that additives facilitate hydrogen bond formation [53,54]. However, at high CD concentrations, this peak red-shifted, with the pure CD group reaching the lowest wavenumber at 1664 cm^−1^. This aligns with the rheological results, suggesting that an excessive CD concentration may lead to structural instability.

### 3.7. SEM Analysis

**Figure 9:**
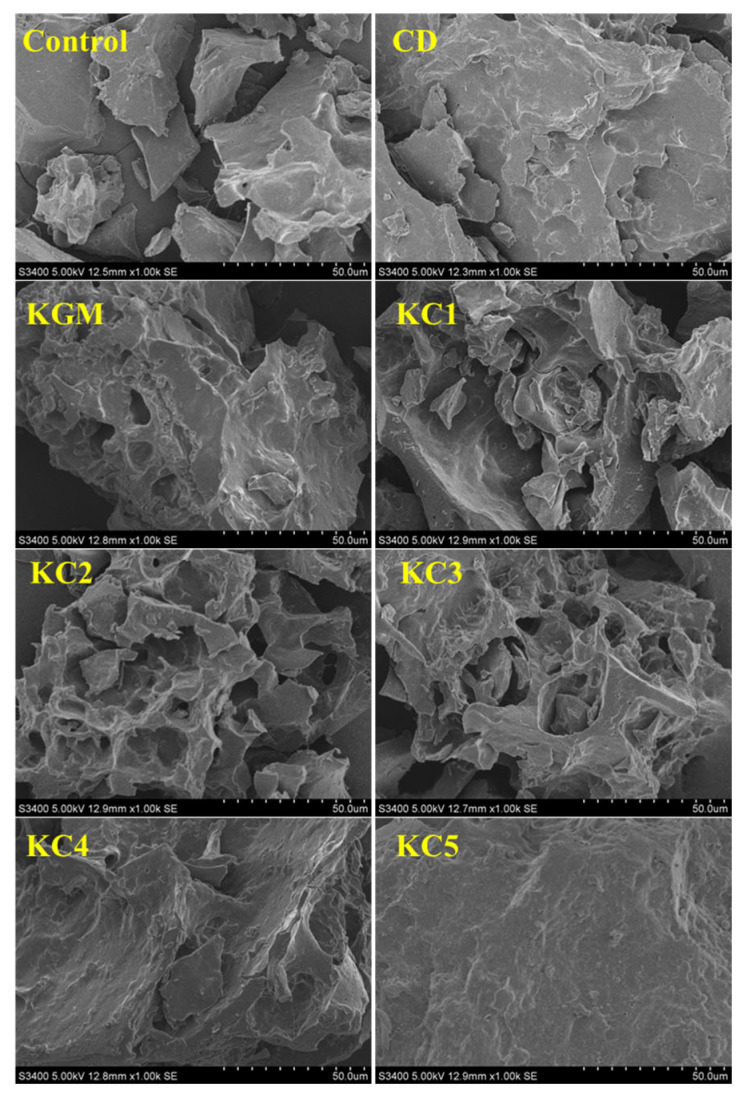
Microstructural characteristics of GBR gel with different formulations at 1000× magnification. The meanings of the symbols for gel formulations are detailed in Table 1.

The microscopic structures of the GBR gels with different formulations at 1000× magnification are shown in Figure 9. The type and ratio of hydrocolloids significantly affected the microstructure of the GBR gels. In the control group, starch molecules are randomly distributed throughout the gel matrix. However, after adding hydrocolloids, the starch molecules form a more ordered structure, which is attributed to the strong interactions between hydrocolloids and starch granules [55]. In the pure CD group, starch molecules gradually aggregate into sheet-like structures. With the addition of KGM, polysaccharides interact closely with water, and after freeze-drying, water separates from the gel matrix, leading to the formation of a porous structure [56]. The KGM group exhibits a rough gel surface, likely due to its high adhesive properties, which allow it to form a sol in the aqueous phase and exhibit strong hydrophilicity, thickening ability, and viscosity [57]. In KC2 and KC3, a distinct honeycomb-like network structure is observed. As the curdlan (CD) content increases, the gel surface becomes smoother and more uniform. However, excessive CD content gradually disrupts and dissolves the honeycomb structure, indicating a weakened hydrogen bonding effect, which in turn reduces the mechanical strength and leads to an unstable sample structure. These findings were consistent with the 3D printability and texture analysis results in Section 3.1 and Section 3.3.

## 4. Conclusions

This study explored the effects of KGM and CD on the 3D printing performance and physicochemical properties of GBR gel. The KGM/CD composite system significantly improved the printing accuracy, shape retention, and rheological stability of GBR gel, enhancing its processing adaptability. Among the tested formulations, the KC3 group (2.25% KGM, 0.75% CD) exhibited the best printing stability and rheological properties. Additionally, the composite system enhanced GBR gel’s hardness, gumminess, cohesiveness, and adhesiveness, providing superior physical characteristics for food 3D printing. Further analysis revealed that the KGM/CD combination strengthened the gel network, improved the water retention capacity, and optimized the microstructural properties, thereby enhancing the printability and rheological performance of the GBR gel. This study demonstrated that GBR has great potential for food 3D printing applications, offering a new approach to the intelligent manufacturing of germinated grains. However, challenges such as incomplete post-printing curing and long-term storage stability still need to be addressed. Future research could focus on optimizing the composite system and incorporating post-processing techniques (e.g., baking, microwave treatment, freeze–thaw, adjusting the pH value of the gel system) to improve the quality and edibility of the printed food. Additionally, a deeper investigation into the effects of KGM/CD combinations on starch retrogradation and storage stability could provide comprehensive theoretical support for the industrial application of food 3D printing technology.

## Figures and Tables

**Table 1:** Experimental formulations of the GBR gel for 3D printing.

Formulations	Hydrocolloids	
	KGM (wt/wt, %)	CD (wt/wt, %)
GBR powder (Control)	0	0
Konjac glucomannan (KGM)	3	0
Curdlan (CD)	0	3
KGM to CD ratio of 1:1 (KC1)	1.5	1.5
KGM to CD ratio of 2:1 (KC2)	2	1
KGM to CD ratio of 3:1 (KC3)	2.25	0.75
KGM to CD ratio of 1:2 (KC4)	1	2
KGM to CD ratio of 1:3 (KC5)	0.75	2.25

Note: The concentrations of KGM and CD (wt/wt, %) were calculated relative to the total weight of GBR powder and water, excluding the hydrocolloids themselves.

**Table 2:** The 3D printing performance of GBR gel with different formulations.

Formulations	*D_L_* (%)	*D_W_* (%)	*D_H_* (%)	*D_C_* (%)	Top	Side
Control	17.08 ± 1.30 ^a^	20.60 ± 0.57 ^a^	16.43 ± 2.88 ^a^	18.04 ± 0.72 ^a^	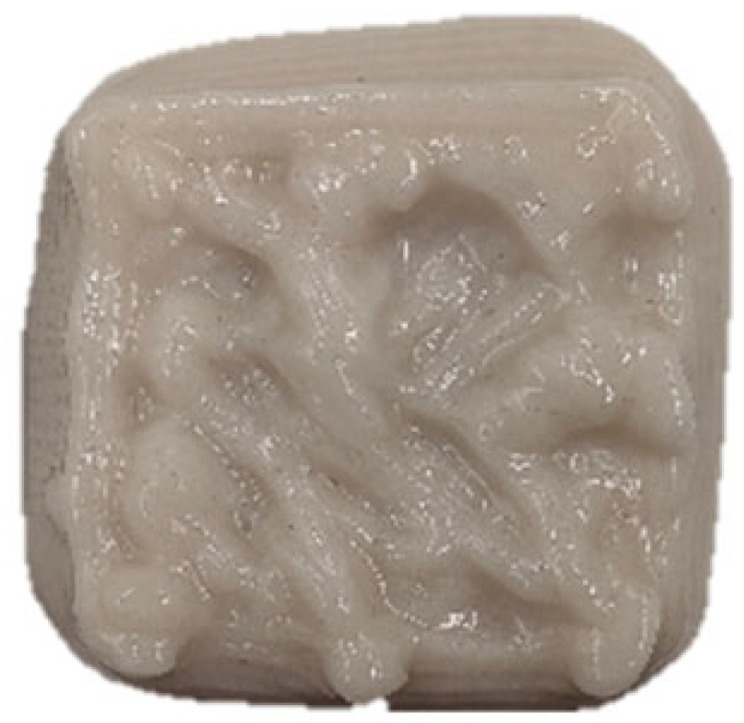	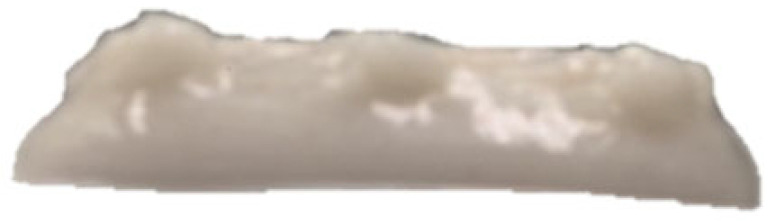
KGM	9.09 ± 0.38 ^b^	10.59 ± 0.032 ^b^	16.23 ± 2.17 ^a^	11.97 ± 0.89 ^b^	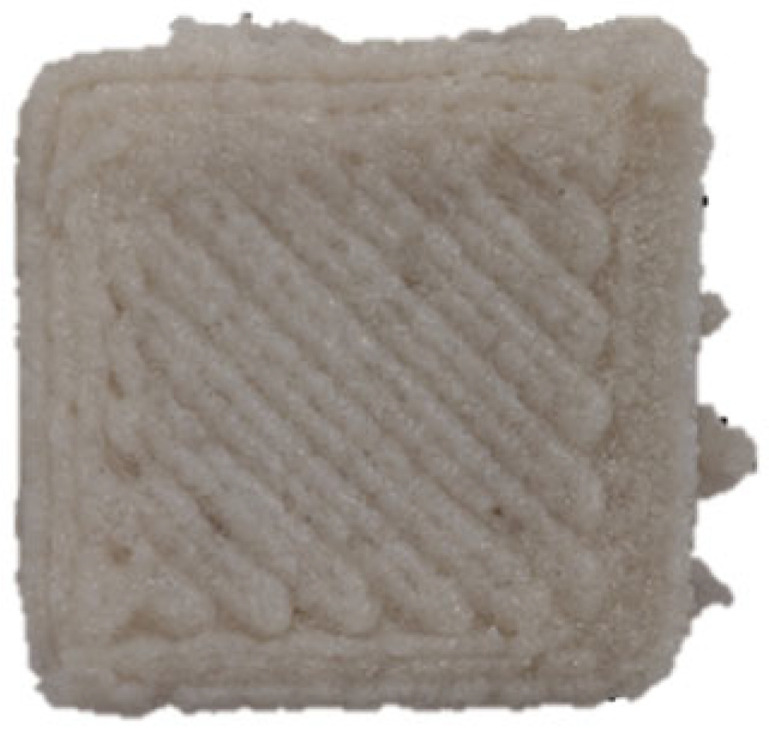	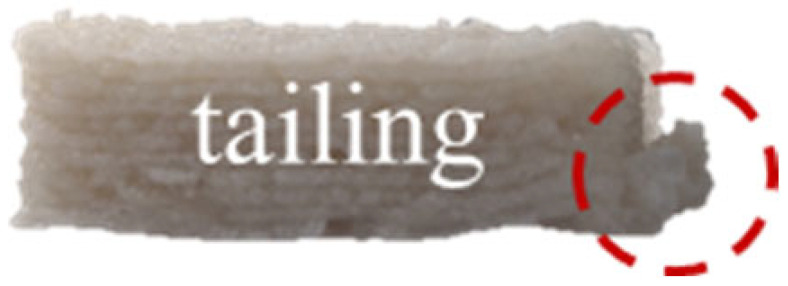
CD	7.77 ± 0.94 ^cd^	4.87 ± 0.25 ^e^	13.23 ± 2.05 ^b^	8.61 ± 0.68 ^d^	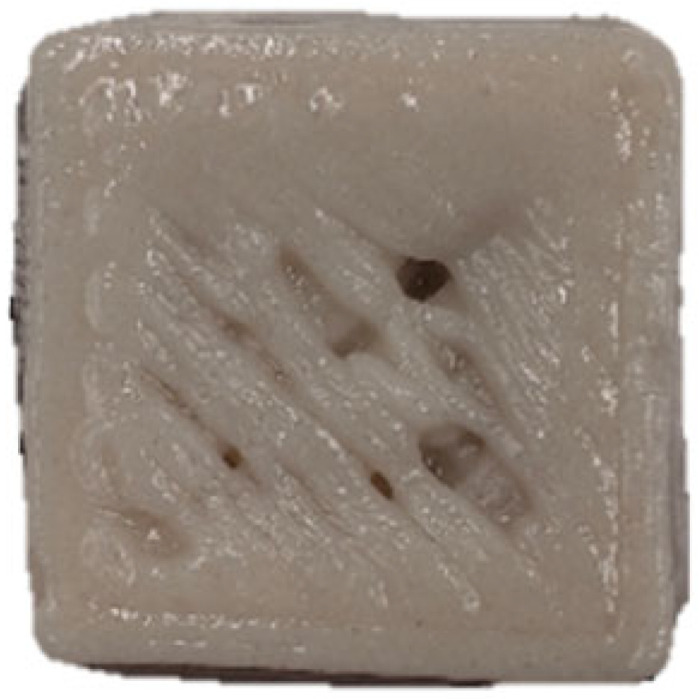	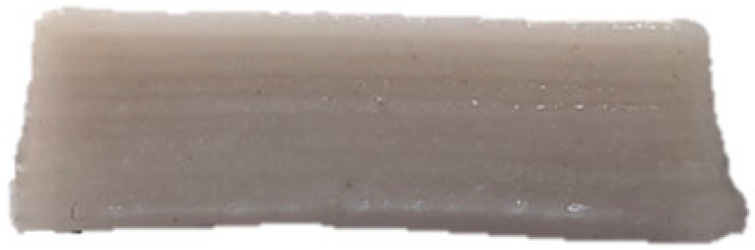
KC1	8.78 ± 0.69 ^bc^	10.12 ± 0.25 ^b^	8.50 ± 0.92 ^de^	9.13 ± 0.33 ^d^	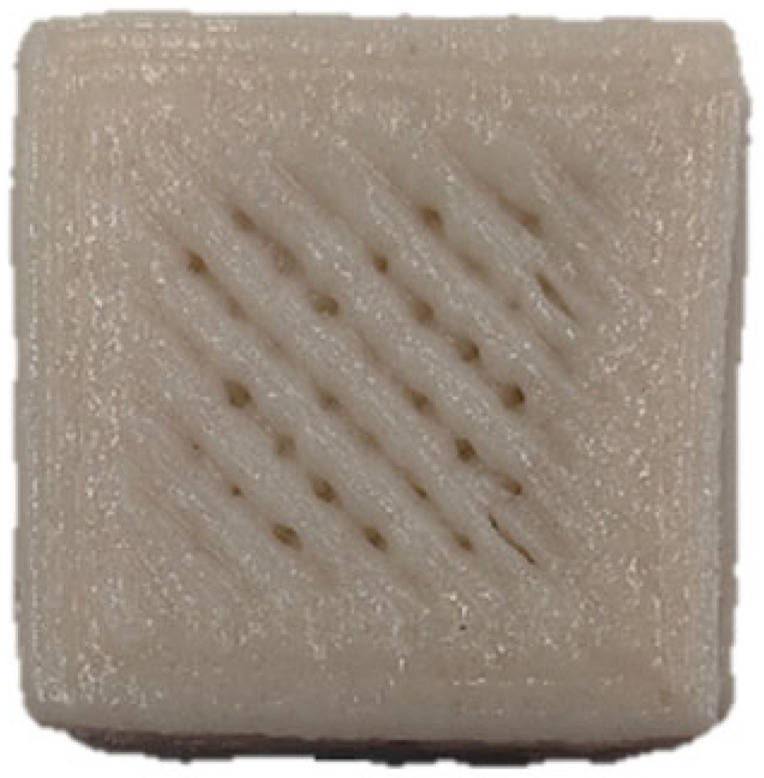	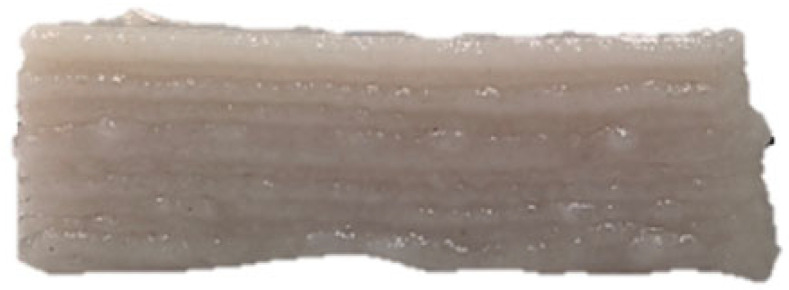
KC2	6.41 ± 0.93 ^e^	6.32 ± 0.10 ^d^	7.17 ± 0.06 ^ef^	6.63 ± 0.65 ^e^	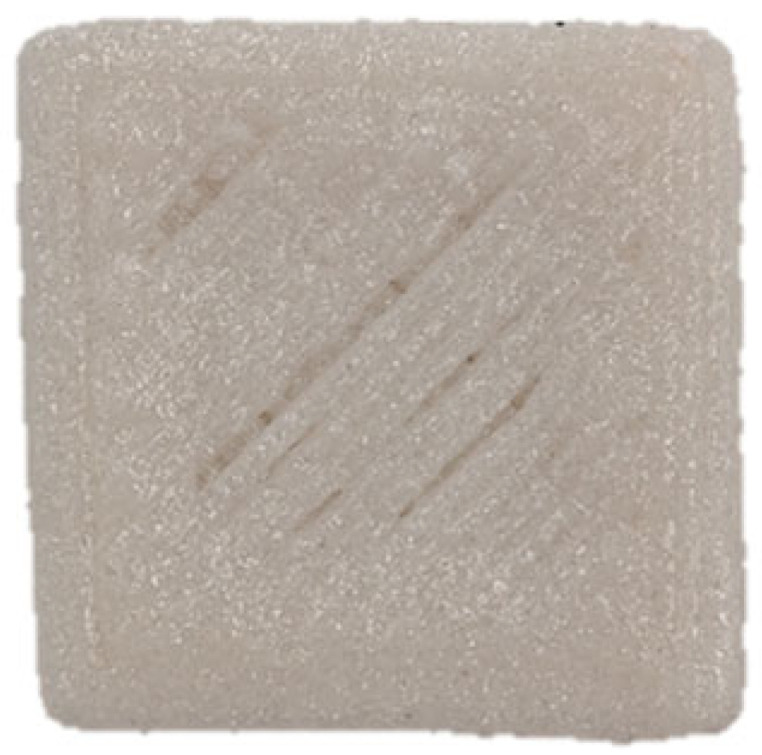	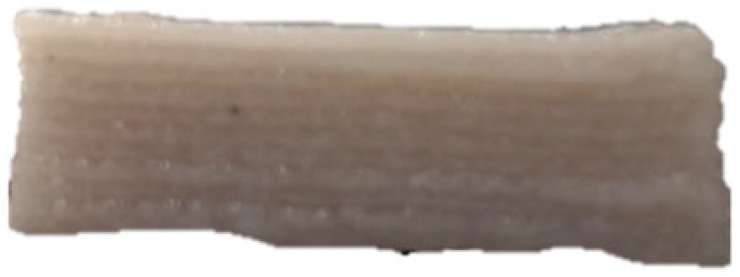
KC3	4.94 ± 0.11 ^e^	4.64 ± 0.29 ^e^	5.30 ± 0.98 ^f^	4.97 ± 0.45 ^f^	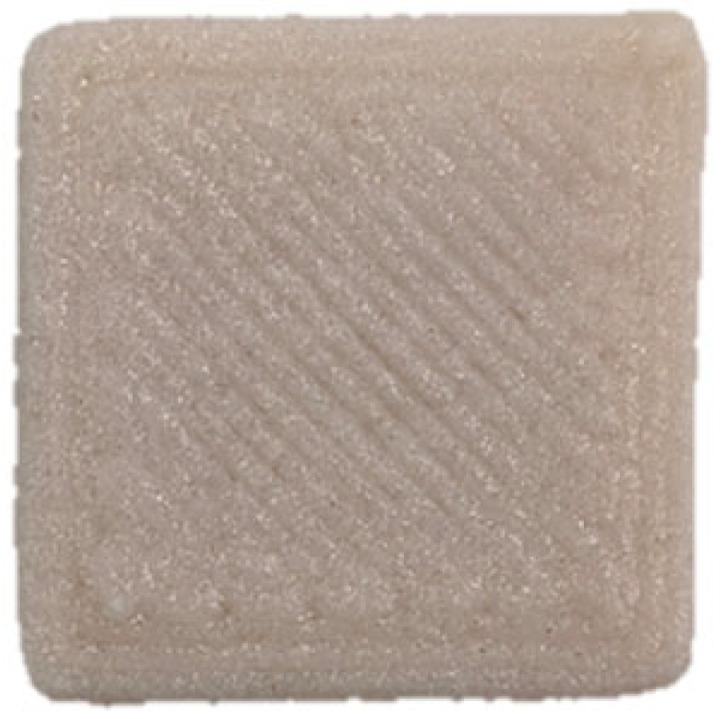	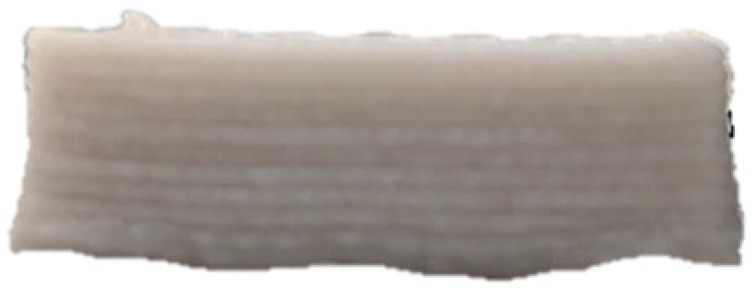
KC4	9.36 ± 0.08 ^b^	10.04 ± 0.32 ^b^	11.23 ± 1.70 ^bc^	10.21 ± 0.56 ^c^	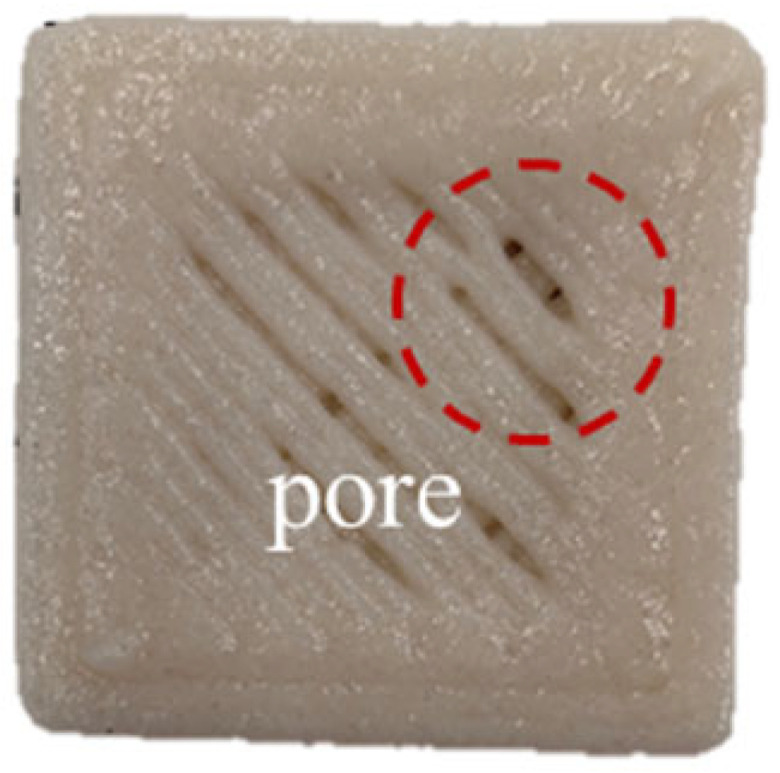	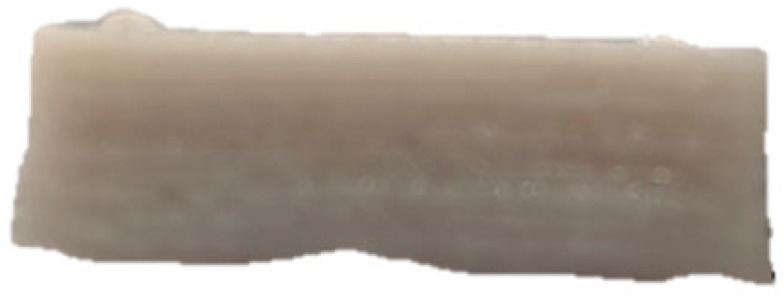
KC5	7.39 ± 0.14 ^de^	7.60 ± 0.55 ^c^	10.37 ± 0.60 ^cd^	8.45 ± 0.13 ^d^	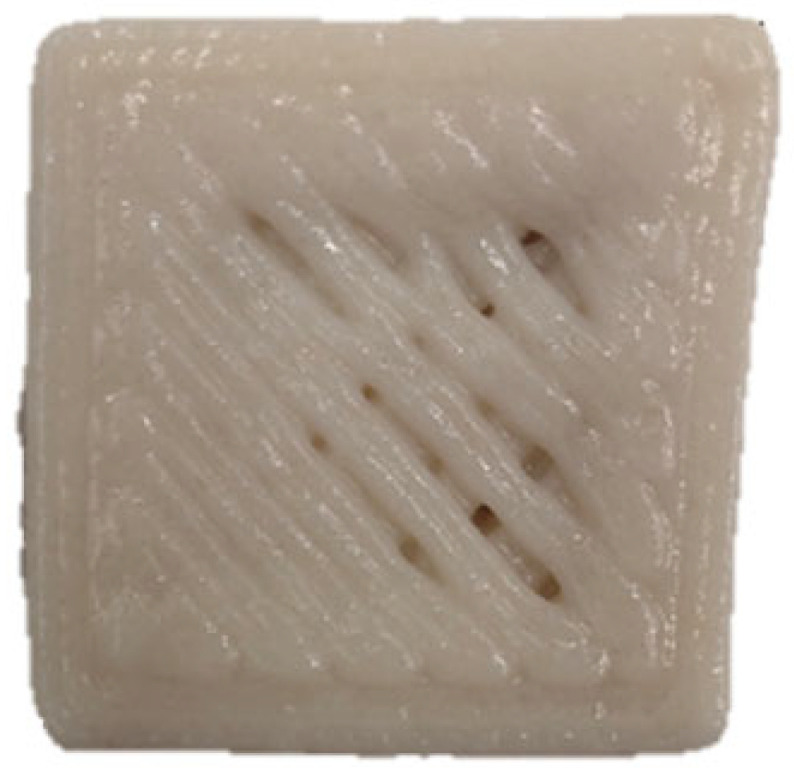	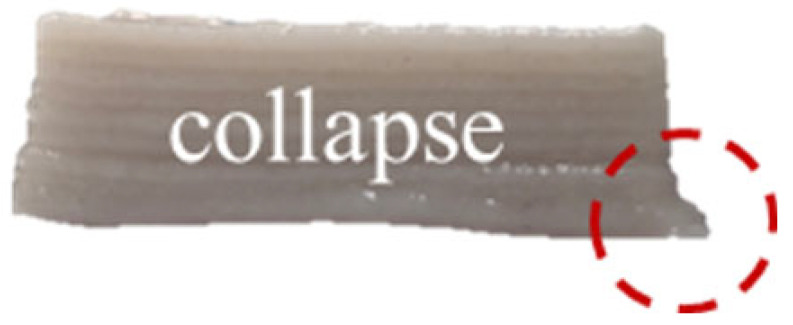

Note: Different letters in the same column indicate significant differences between samples (*p* < 0.05). The red circle represents the undesired printing phenomena (such as pore, tailing, collapse, etc.) compared to the GBR gel model shown in Figure 1b.

**Table 3:** Power-law model parameters of GBR gel.

Formulation	Fitted Parameters of Power-Law Model		
	*K* (Pa·s)	*n*	*R* ^2^
Control	126.27 ± 5.56 ^e^	0.051 ± 0.001 ^c^	0.997
KGM	804.12 ± 35.36 ^a^	0.061 ± 0.001 ^c^	0.998
CD	242.16 ± 9.88 ^d^	0.163 ± 0.023 ^ab^	0.956
KC1	397.61 ± 5.34 ^b^	0.049 ± 0.014 ^c^	0.992
KC2	389.61 ± 20.83 ^b^	0.152 ± 0.015 ^b^	0.991
KC3	384.04 ± 53.72 ^bc^	0.151 ± 0.001 ^b^	0.986
KC4	344.54 ± 23.39 ^c^	0.179 ± 0.014 ^a^	0.983
KC5	216.34 ± 36.02 ^d^	0.157 ± 0.025 ^ab^	0.974

Note: *γ*, shear rate; *K*, consistency coefficient; *n*, flow behavior index. Different letters in the same column indicate significant differences between samples (*p* < 0.05). The meanings of the symbols for gel formulations are detailed in Table 1.

## Data Availability

The original contributions presented in the study are included in the article, further inquiries can be directed to the corresponding authors.

## References

[1] Kuo C.-C., Liu L.-C., Teng W.-F., Chang H.-Y., Chien F.-M., Liao S.-J., Kuo W.-F., Chen C.-M. (2016). Preparation of Starch/Acrylonitrile-Butadiene-Styrene Copolymers (ABS) Biomass Alloys and Their Feasible Evaluation for 3D Printing Applications. Compos. Part B Eng..

[2] Feng C., Zhang M., Bhandari B., Wang Y., Wang B. (2021). Improvement of 3D Printing Properties of Rose-Sodium Alginate Heterogeneous Gel by Adjusting Rose Material. J. Food Process Eng..

[3] Cheng Y., Yuqing H., Xiao L., Gao W., Kang X., Sui J., Cui B. (2024). Impact of Starch Amylose and Amylopectin on the Rheological and 3D Printing Properties of Cornstarch. Int. J. Biol. Macromol..

[4] Shen L., Zhu Y., Liu C., Wang L., Liu H., Kamruzzaman M., Liu C., Zhang Y., Zheng X. (2020). Modelling of Moving Drying Process and Analysis of Drying Characteristics for Germinated Brown Rice under Continuous Microwave Drying. Biosyst. Eng..

[5] Kim H.W., Bae H., Park H.J. (2018). Reprint of: Classification of the Printability of Selected Food for 3D Printing: Development of an Assessment Method Using Hydrocolloids as Reference Material. J. Food Eng..

[6] Patil S.B., Khan M.K. (2011). Germinated Brown Rice as a Value Added Rice Product: A Review. J. Food Sci. Technol..

[7] Hou D., Tang J., Feng Q., Niu Z., Shen Q., Wang L., Zhou S. (2024). Gamma-Aminobutyric Acid (GABA): A Comprehensive Review of Dietary Sources, Enrichment Technologies, Processing Effects, Health Benefits, and Its Applications. Crit. Rev. Food Sci. Nutr..

[8] Zhu H., Liu C., Bai C., Chen Q., Zhao X., Liu C., Zheng X., Shen L. (2024). Effects of Microwave Vacuum Drying on Drying Characteristics, Quality Attributes and Starch Structure of Germinated Brown Rice. Int. J. Biol. Macromol..

[9] Ji S., Xu T., Li Y., Li H., Zhong Y., Lu B. (2022). Effect of Starch Molecular Structure on Precision and Texture Properties of 3D Printed Products. Food Hydrocoll..

[10] Cheng Y., Chen Y., Gao W., Kang X., Sui J., Yu B., Guo L., Zhao M., Yuan C., Cui B. (2024). Investigation of the Mechanism of Gelatin to Enhance 3D Printing Accuracy of Corn Starch Gel: From Perspective of Phase Morphological Changes. Int. J. Biol. Macromol..

[11] Ma S., Liu J., Zhang Q., Lin Q., Liu R., Xing Y., Jiang H. (2022). 3D Printing Performance Using Radio Frequency Electromagnetic Wave Modified Potato Starch. Innov. Food Sci. Emerg. Technol..

[12] Shi S., Wen J., Geng H., Zhan X., Liu Y. (2024). Physicochemical Properties, Structural Properties and Gels 3D Printing Properties of Wheat Starch. Int. J. Biol. Macromol..

[13] Ji S., Xu M., Li Y., Zou Y., Zhou Z., Zhao X., Shen J., Lu B. (2025). Exploring the Mechanism of Fatty Acids to Improve 3D Printing Precision of Cassava Starch Gel. Food Res. Int..

[14] Tian H., Wang K., Lan H., Wang Y., Hu Z., Zhao L. (2021). Effect of Hybrid Gelator Systems of Beeswax-Carrageenan-Xanthan on Rheological Properties and Printability of Litchi Inks for 3D Food Printing. Food Hydrocoll..

[15] Yu J., Li D., Wang L., Wang Y. (2023). Improving Freeze-Thaw Stability and 3D Printing Performance of Soy Protein Isolate Emulsion Gel Inks by Guar & Xanthan Gums. Food Hydrocoll..

[16] Yu X., Wang Y., Zhao W., Li S., Pan J., Prakash S., Dong X. (2023). Hydrophilic Colloids (Konjac Gum/Xanthan Gum) in 3D Printing of Transitional Food from Fish Paste. Food Hydrocoll..

[17] Jiang Y., Reddy C.K., Huang K., Chen L., Xu B. (2019). Hydrocolloidal Properties of Flaxseed Gum/Konjac Glucomannan Compound Gel. Int. J. Biol. Macromol..

[18] Zhang R., Edgar K.J. (2014). Properties, Chemistry, and Applications of the Bioactive Polysaccharide Curdlan. Biomacromolecules.

[19] Kim J., Kim J.S., Moon K.-D. (2025). 3D-Printed Rice Cake for Dysphagia Diet: Effect of Rice Flour/κ-Carrageenan/Curdlan Complex Gel on Structure, Swallowability, and Storage. Future Foods.

[20] Feng M., Zhang M., Mujumdar A.S., Guo Z. (2024). Influence of Components Interaction in Recombined Food Gels on 3D Printing: A Comprehensive Review. Food Hydrocoll..

[21] Wu C., Peng S., Wen C., Wang X., Fan L., Deng R., Pang J. (2012). Structural Characterization and Properties of Konjac Glucomannan/Curdlan Blend Films. Carbohydr. Polym..

[22] Huang M., Zhang M., Guo C. (2020). 3D Printability of Brown Rice Gel Modified by Some Food Hydrocolloids. J. Food Process. Preserv..

[23] He A., Xu J., Hu Q., Zhao L., Ma G., Zhong L., Liu R. (2023). Effects of Gums on 3D Printing Performance of Pleurotus Eryngii Powder. J. Food Eng..

[24] Cao Y., Zhang F., Guo P., Dong S., Li H. (2019). Effect of Wheat Flour Substitution with Potato Pulp on Dough Rheology, the Quality of Steamed Bread and in Vitro Starch Digestibility. LWT.

[25] Park B.-R., No J., Oh H., Park C.S., You K.-M., Chewaka L.S. (2025). Exploring Puffed Rice as Anovel Ink for 3D Food Printing: Rheological Characterization and Printability Analysis. J. Food Eng..

[26] Karppinen A., Saarinen T., Salmela J., Laukkanen A., Nuopponen M., Seppälä J. (2012). Flocculation of Microfibrillated Cellulose in Shear Flow. Cellulose.

[27] Wang M., Chen C., Sun G., Wang W., Fang H. (2010). Effects of Curdlan on the Color, Syneresis, Cooking Qualities, and Textural Properties of Potato Starch Noodles. Starch-Stärke.

[28] Nakao Y. (1991). Curdlan: Properties and Application to Foods. Nippon Shokuhin Kogyo Gakkaishi.

[29] Wang S., Wu Z., Jia L., Wang X., He T., Wang L., Yao G., Xie F. (2024). Soybean Protein Isolate-Sodium Alginate Double Network Emulsion Gels: Mechanism of Formation and Improved Freeze-Thaw Stability. Int. J. Biol. Macromol..

[30] Koo C.K.W., Chung C., Fu J.-T.R., Sher A., Rousset P., McClements D.J. (2019). Impact of Sodium Caseinate, Soy Lecithin and Carrageenan on Functionality of Oil-in-Water Emulsions. Food Res. Int..

[31] 3Singh A., Geveke D.J., Yadav M.P. (2017). Improvement of Rheological, Thermal and Functional Properties of Tapioca Starch by Using Gum Arabic. LWT.

[32] Ma S., Zhu P., Wang M. (2019). Effects of Konjac Glucomannan on Pasting and Rheological Properties of Corn Starch. Food Hydrocoll..

[33] Wedamulla N.E., Fan M., Choi Y.-J., Kim E.-K. (2023). Combined Effect of Heating Temperature and Content of Pectin on the Textural Properties, Rheology, and 3D Printability of Potato Starch Gel. Int. J. Biol. Macromol..

[34] Diañez I., Gallegos C., Brito-de la Fuente E., Martínez I., Valencia C., Sánchez M.C., Franco J.M. (2021). Implementation of a Novel Continuous Solid/Liquid Mixing Accessory for 3D Printing of Dysphagia-Oriented Thickened Fluids. Food Hydrocoll..

[35] Liu Z., Bhandari B., Prakash S., Mantihal S., Zhang M. (2019). Linking Rheology and Printability of a Multicomponent Gel System of Carrageenan-Xanthan-Starch in Extrusion Based Additive Manufacturing. Food Hydrocoll..

[36] Wang M., Li D., Zang Z., Sun X., Tan H., Si X., Tian J., Teng W., Wang J., Liang Q. (2022). 3D Food Printing: Applications of Plant-Based Materials in Extrusion-Based Food Printing. Crit. Rev. Food Sci. Nutr..

[37] Pan Y., Sun Q., Liu Y., Wei S., Xia Q., Zheng O., Liu S., Ji H., Deng C., Hao J. (2021). The Relationship between Rheological and Textural Properties of Shrimp Surimi Adding Starch and 3D Printability Based on Principal Component Analysis. Food Sci. Nutr..

[38] Oishi Y., Udagawa H., Shinozaki Y., Moriyama M., Taniguchi H., Kobayashi-Hattori K., Arai S., Takita T. (2009). Preparation of Hypoallergenic Wheat Flour Noodles and Evaluation of Their Physical Properties. Food Sci. Technol. Res..

[39] Wu C., Yuan C., Chen S., Liu D., Ye X., Hu Y. (2015). The Effect of Curdlan on the Rheological Properties of Restructured Ribbonfish (*Trichiurus* spp.) Meat Ge. Food Chem..

[40] Zhao N., Guo C., Liu Z., Chen L., Hu Y., Han M., Huang F., Kang Z., Feng X. (2024). Effects of Different Hydrocolloids on the 3D Printing and Thermal Stability of Chicken Paste. Int. J. Biol. Macromol..

[41] Han N.-R., Bae J.-E., An H.W., Yun H.-J., Retnoaji B., Lee S., Lee S.G. (2025). Influence of Potato Starch on the 3D Printing of Senior-Friendly Foods Enriched with Oyster Powder. LWT.

[42] Liu Z., Zhang M., Bhandari B., Yang C. (2018). Impact of Rheological Properties of Mashed Potatoes on 3D Printing. J. Food Eng..

[43] Zheng Z., Zhang M., Liu Z. (2021). Investigation on Evaluating the Printable Height and Dimensional Stability of Food Extrusion-Based 3D Printed Foods. J. Food Eng..

[44] Phuhongsung P., Zhang M., Devahastin S. (2020). Investigation on 3D Printing Ability of Soybean Protein Isolate Gels and Correlations with Their Rheological and Textural Properties via LF-NMR Spectroscopic Characteristics. LWT.

[45] Zhang H., Xiong Y., Bakry A.M., Xiong S., Yin T., Zhang B., Huang J., Liu Z., Huang Q. (2019). Effect of Yeast β-Glucan on Gel Properties, Spatial Structure and Sensory Characteristics of Silver Carp Surimi. Food Hydrocoll..

[46] Liu Y., Sun Q., Wei S., Xia Q., Pan Y., Liu S., Ji H., Deng C., Hao J. (2022). LF-NMR as a Tool for Predicting the 3D Printability of Surimi-Starch Systems. Food Chem..

[47] Yamul D.K., Navarro A.S. (2020). Effect of Hydrocolloids on Structural and Functional Properties of Wheat/Potato (50/50) Flour Dough. Food Struct..

[48] Joshi R., Baek I., Joshi R., Kim M.S., Cho B.-K. (2022). Detection of Fabricated Eggs Using Fourier Transform Infrared (FT-IR) Spectroscopy Coupled with Multivariate Classification Techniques. Infrared Phys. Technol..

[49] Meng F., Zheng L., Wang Y., Liang Y., Zhong G. (2014). Preparation and Properties of Konjac Glucomannan Octenyl Succinate Modified by Microwave Method. Food Hydrocoll..

[50] Zhou T.-Q., Wang X.-C., Gao L.-Y., Yan J.-N., Wu H.-T. (2024). Construction and Properties of Curdlan Gum/Gellan Gum Binary Composite Gel System. Food Hydrocoll..

[51] Wu K., Wan Y., Li X., Qian H., Xiao M., Ni X., Jiang F., Chen S. (2021). Impact of Heating and Drying Temperatures on the Properties of Konjac Glucomannan/Curdlan Blend Films. Int. J. Biol. Macromol..

[52] Xing X., Chitrakar B., Hati S., Xie S., Li H., Li C., Liu Z., Mo H. (2022). Development of Black Fungus-Based 3D Printed Foods as Dysphagia Diet: Effect of Gums Incorporation. Food Hydrocoll..

[53] Huang J., Zhang M., Mujumdar A.S., Li C. (2024). Modulation of Starch Structure, Swallowability and Digestibility of 3D-Printed Diabetic-Friendly Food for the Elderly by Dry Heating. Int. J. Biol. Macromol..

[54] Liu L., Xie T., Cheng W., Ding Y., Xu B. (2024). Characterization and Mechanism of Thermally Induced Tea Polyphenols. LWT.

[55] Hu W., Gu J., Yang K., Bu T., Natallia K., Zhang Z., Wu W. (2025). Mechanism Ofhydrocolloids Effect Onbuckwheat Starch Gelsfrom Interaction Andstructural Perspectives: Acomparative Study. Int. J. Biol. Macromol..

[56] Song J., Rong L., Li J., Shen M., Yu Q., Chen Y., Kong J., Xie J. (2024). Effects of Three Different Polysaccharides on the Sol Gel-Behavior, Rheological, and Structural Properties of Tapioca Starch. Int. J. Biol. Macromol..

[57] Lu C., Guo J., Li P., Bai Z., Cui G., Li P. (2025). Physicochemical Properties and Invitro Digestion Ofquinoa Starch Induced by Combination of Ultrasound and Konjac Glucomannan. Food Chem..

